# The CHARTER-Ireland trial: can nebulised heparin reduce acute lung injury in patients with SARS-CoV-2 requiring advanced respiratory support in Ireland: a study protocol and statistical analysis plan for a randomised control trial

**DOI:** 10.1186/s13063-022-06518-z

**Published:** 2022-09-14

**Authors:** John Robert Sheehan, Padraig Calpin, Maeve Kernan, Claire Kelly, Siobhan Casey, Darragh Murphy, Alberto Alvarez-Iglesias, Camilla Giacomini, Catriona Cody, Gerard Curley, Shane McGeary, Ciara Hanley, Bairbre McNicholas, Frank van Haren, John G. Laffey, David Cosgrave

**Affiliations:** 1 University Hospital Galway, Newcastle Road, Galway, Ireland; 2 National University of Ireland Galway, Galway, Ireland; 3 Connolly Hospital, Blanchardstown, Dublin 15, Ireland; 4 Beaumont Hospital, Beaumont, Dublin, Ireland; 5 St George Hospital, Sydney, Australia

**Keywords:** COVID-19, CHARTER, Acute respiratory distress syndrome, SARS-CoV-2, Respiratory support, Nebulised heparin

## Abstract

**Background:**

COVID-19 pneumonia is associated with the development of acute respiratory distress syndrome (ARDS) displaying some typical histological features. These include diffuse alveolar damage with extensive pulmonary coagulation activation. This results in fibrin deposition in the microvasculature, leading to the formation of hyaline membranes in the air sacs. Well-conducted clinical trials have found that nebulised heparin limits pulmonary fibrin deposition, attenuates progression of ARDS, hastens recovery and is safe in non-COVID ARDS. Unfractionated heparin also inactivates the SARS-CoV-2 virus and prevents entry into mammalian cells. Nebulisation of heparin may therefore limit fibrin-mediated lung injury and inhibit pulmonary infection by SARS-CoV-2. Based on these findings, we designed the CHARTER-Ireland Study, a phase 1b/2a randomised controlled study of nebulised heparin in patients requiring advanced respiratory support for COVID-19 pneumonia.

**Methods:**

This is a multi-centre, phase 1b/IIa, randomised, parallel-group, open-label study. The study will randomise 40 SARs-CoV-2-positive patients receiving advanced respiratory support in a critical care area. Randomisation will be via 1:1 allocation to usual care plus nebulised unfractionated heparin 6 hourly to day 10 while receiving advanced respiratory support or usual care only. The study aims to evaluate whether unfractionated heparin will decrease the procoagulant response associated with ARDS up to day 10. The study will also assess safety and tolerability of nebulised heparin as defined by number of severe adverse events; oxygen index and respiratory oxygenation index of intubated and unintubated, respectively; ventilatory ratio; and plasma concentration of interleukin (IL)-1β, IL6, IL-8, IL-10 and soluble tumour necrosis factor receptor 1, C-reactive protein, procalcitonin, ferritin, fibrinogen and lactate dehydrogenase as well as the ratios of IL-1β/IL-10 and IL-6/IL-10. These parameters will be assessed on days 1, 3, 5 and 10; time to separation from advanced respiratory support, time to discharge from the intensive care unit and number tracheostomised to day 28; and survival to days 28 and 60 and to hospital discharge, censored at day 60. Some clinical outcome data from our study will be included in the international meta-trials, CHARTER and INHALE-HEP.

**Discussion:**

This trial aims to provide evidence of potential therapeutic benefit while establishing safety of nebulised heparin in the management of ARDS associated with SARs-CoV-2 infection.

**Trial registration:**

ClinicalTrials.govNCT04511923. Registered on 13 August 2020.

Protocol version 8, 22/12/2021

Protocol identifier: NUIG-2020-003

EudraCT registration number: 2020-003349-12 9 October 2020

**Supplementary Information:**

The online version contains supplementary material available at 10.1186/s13063-022-06518-z.

## Administrative information

‘Note: the numbers in curly brackets in this protocol refer to SPIRIT checklist item numbers. The order of the items has been modified to group similar items (see http://www.equator-network.org/reporting-guidelines/spirit-2013-statement-defining-standard-protocol-items-for-clinical-trials).’

**Table:** 

Title {1}	The CHARTER-Ireland Trial: Can nebulised heparin reduce acute lung injury in patients with SARS-CoV-2 requiring advanced respiratory support in Ireland: a study protocol and statistical analysis plan for a randomised control trial
Trial registration {2a and 2b}.	ClinicalTrials.gov, ID: NCT04511923. Registered on 13 August 2020. EudraCT registration number: 2020-003349-12 9 October 2020
Protocol version {3}	Protocol Version 8, 22 December 2022 Protocol Identifier: NUIG-2020-003
Funding {4}	Funding for this study is from CURAM/SFI and Aerogen.
Author details {5a}	Dr. John Robert Sheehan, University Hospital Galway Dr. Padraig Calpin, University Hospital Galway Siobhan Casey, University Hospital Galway Maeve Kernan, National University of Ireland Galway Claire Kelly, National University of Ireland Galway Darragh Murphy, National University of Ireland Galway Alberto Alvarez-Iglesias, National University of Ireland Galway Dr Camilla Giacomini, University Hospital Galway Dr Catriona Cody James Connolly Hospital, Blanchardstown Prof Gerard Curley, Royal College of Surgeons in Ireland, Beaumont Hospital, Dublin. Dr Shane McGeary, James Connolly Hospital, Blanchardstown, Dr Ciara Hanley, University Hospital Galway Dr Bairbre McNicholas, University Hospital Galway, National University of Ireland Galway. Prof. Frank van Haren, St George Hospital, Sydney, College of Health and Medicine, Australian National University Prof. John G. Laffey, University Hospital Galway, National University of Ireland Galway. Dr. David Cosgrave, University Hospital Galway, National University of Ireland Galway.
Name and contact information for the trial sponsor {5b}	Prof. Jim Livesey, (Vice President of Research) National University of Ireland Galway University Road Galway (091) 524411.
Role of sponsor {5c}	The National University of Ireland, Galway is the study sponsor and is responsible for oversight of the study, and ensuring compliance with GCP, clinical trials legislation and the relevant ethical approval.

## Introduction

### Background and rationale {6a}

SARS-CoV-2 generates a robust immune response, with inflammatory cytokine generation and excessive coagulation activation [1]. Excess coagulation leads to increased fibrin formation, resulting in deposition of hyaline membranes in the alveolar spaces and microvascular thrombi, contributing to the development of ARDS and multiorgan failure [1, 2]. A rise in d-dimer level associated with the extent of fibrin formation correlates well with the severity of ARDS [1]. Lan et al. showed in their meta-analysis that nebulised heparin can be of benefit in burn injury to the lung, a condition associated with procoagulant and inflammatory effects also [3]. Further laboratory research by Camprubí-Rimblas et al. examined the in vitro effects of heparin on a cellular model of ARDS, showing a reduction in pro-inflammatory markers in heparin-treated cells [4].

There is scientific rationale for the use of nebulised heparin in the treatment of SARS-CoV-2 also [5]. Dixon et al. found secondary outcome evidence of benefit through reduction in the Murray Lung Injury Score and reduction in the development of ARDS along with improving outcomes at day 60 in mechanically ventilated patients at risk of developing ARDS who received nebulised heparin. However, the primary outcome of improvement in physical function score and mortality were not different [6]. This study gives further indication that nebulised heparin may limit fibrin deposition and could help to prevent progression of ARDS [6]. Furthermore, SARS-CoV-2 spike glycoprotein has been found to bind heparin, causing a conformational change which prevents the virus from entering the cell [7].

This is a preliminary efficacy and safety study, as such d-dimer level is being used as an intermediate marker of efficacy. Higher d-dimer levels are correlated with more severe ARDS in SARS-CoV-2 infection [8]. Lower mean d-dimer levels were found in survivors of SARS-CoV-2 vs non-survivors [9]. This study aims to test the hypothesis that nebulised heparin will cause a reduction in pulmonary microvascular thrombosis, using a reduction in d-dimer as a surrogate marker. Recent studies using nebulised heparin have not shown an increased rate of adverse outcomes when compared with placebo, and this study aims to confirm these findings [6, 10].

A number of previous clinical trials in patients with, or at risk for, ARDS or with related conditions, report that nebulised heparin is safe, and may have therapeutic potential, limiting pulmonary fibrin deposition, reducing acute lung injury progression and facilitating recovery. Furthermore, the potential exists for heparin to directly inactivate SARS-CoV-2. Given these findings, we believe that a safety and feasibility study of nebulised heparin therapy for the treatment of COVID-19 is warranted.

### Objectives {7}

Our hypothesis is that nebulised heparin will decrease severity of lung injury in SARS-CoV-2 through multiple mechanisms as shown above, and this will be shown through a decrease in procoagulant markers such as d-dimer.

The objective of this safety and feasibility study is to determine if treatment with nebulised heparin, compared to standard care, reduces the severity of the procoagulant response in intensive care unit (ICU) patients with SARS-CoV-2 requiring advanced respiratory support and to assess the safety of nebulised heparin in patients with COVID-19-induced severe respiratory failure.

### Trial design {8}

This is an investigator-led prospective, phase 1b/IIa, multi-centre, randomised, parallel-group, open-label proof-of-principle, superiority study. The study will recruit 40 adult patients infected with SARS-CoV-2 who require advanced respiratory support across multiple centres in Ireland. Randomisation will use a 1:1 allocation to usual care only or usual care plus nebulised unfractionated heparin 6 hourly to day 10 while receiving advanced respiratory support. The study is designed to evaluate whether nebulised unfractionated heparin will decrease the procoagulant state related to ARDS. The study will measure safety outcomes for the administration of heparin via the Aerogen solo nebuliser as a co-primary endpoint and will assess clinical outcomes as secondary endpoints. The study adheres to the SPIRIT guidance on clinical trials [11].

## Methods: participants, interventions and outcomes

### Study setting {9}

Three intensive care units across Ireland were selected as enrolment sites. The size of the intensive care units differed across the 3 hospitals. However, all ICUs adhere to the JFICM/ICSI intensive care guidelines of 1:1 nurse care in level 3 beds, 1:2 nurse care in level 2 beds, 1 nonconsultant hospital doctor per 6–8 patients and a dedicated consultant physician responsible for the care of the patients in the level 2/3 beds.

### Eligibility criteria {10}

Patients with confirmed SARS-CoV-2 infection receiving advanced respiratory support in an intensive care environment. See Table 1.

**Table 1:** Eligibility criteria for participants

Inclusion criteria	Exclusion criteria
Confirmed or suspected COVID-19 If ‘suspected’, results must be pending or testing intended	Enrolled in another clinical trial that is unapproved for co-enrolment
Ability to obtain informed consent/assent to participate in study	Heparin allergy or heparin-induced thrombocytopenia
Age 18 years or older	APTT > 100 s
Requiring high-flow nasal oxygen (>30l/min) or positive pressure ventilator support or invasive mechanical ventilation in the ICU for a time period of no greater than 48 h	Pulmonary bleeding, which is frank bleeding in the trachea, bronchi or lungs with repeated haemoptysis or requiring repeated suctioning
D-dimers > 200 ng/ml	Platelet count < 50 × 10^9^ per l
Acute opacities on chest imaging affecting at least one lung quadrant. ‘Acute opacities’ do not include effusions, lobar/lung collapse or nodules	Uncontrolled bleeding
Currently in a higher level of care area designated for inpatient care of patients where therapies including non-positive pressure ventilatory support (e.g. high-flow nasal oxygen) can be provided	Pregnant or suspected pregnancy (urine or serum HCG will be recorded)
	Receiving or about to commence ECMO or HFOV
	Myopathy, spinal cord injury or nerve injury or disease with a likely prolonged incapacity to breathe independently, e.g. Guillain-Barre syndrome
	Usually receives home oxygen
	Fully dependent on others for personal care due to physical or cognitive decline prior to this illness (premorbid status)
	Death is imminent or inevitable within 24 h
	The clinical team would not be able to set up the study nebuliser and ventilator circuit as required including with active humidification
	Clinician objection
	The use or anticipated use of nebulised tobramycin during this clinical episode
	Any other specific contraindication to anticoagulation including prophylactic anticoagulation not otherwise listed here
	Relapse in clinical condition requiring advances respiratory support in patient that had fully weaned from advanced respiratory support
	Receiving any direct/novel oral anticoagulant

### Who will take informed consent? {26a}

Informed consent is taken by an investigating doctor conducted in accordance with the ethical principles outlined in the Declaration of Helsinki. For trial participants who are unable to give consent due to illness severity, informed assent is obtained from next of kin. Due to the pandemic situation, some modifications to the assent process were required and approval was received from the Irish Health Research Consent Declaration Committee to allow phone assent to be obtained when the next of kin was unable to attend the hospital. If or when these patients regain capacity, their informed consent to continue to participate in the study will then be sought, with this consent then superseding the consent declaration and assent.

### Additional consent provisions for collection and use of participant data and biological specimens {26b}

Provisions for consent/assent to use patient data and biological specimens in future ancillary studies is included in our study protocol and has similar approvals in place.

### Interventions

#### Explanation for the choice of comparators {6b}

As a phase 1b/2a study of a new treatment for this disease, the comparator chosen was standard care in our participants.

#### Intervention description {11a}

Participants assigned to ‘nebulised heparin’ will receive 5ml heparin sodium 5000 I.U./ml (25,000 I.U. in 5 ml), manufactured by Pinewood laboratories, Ballymacarbry, Clonmel, Co. Tipperary. This will be administered via the Aerogen solo nebuliser every 6 h from enrolment to day 10, in addition to the standard care required as determined by the treating team. Participants assigned to ‘standard care’ will receive the standard care required as determined by the treating team and will not be treated with nebulised heparin.

#### Criteria for discontinuing or modifying allocated interventions {11b}

Outlined in Table 2.

**Table 2:** Indications for withholding and recommencement of nebulised heparin

Nebulised heparin should be withheld if any of the following occurs:	Nebulised heparin should be recommenced if:
More than 10 days have elapsed since randomisation	Having been withheld because the patient was outside the ICU, the patient returns to ICU
The patient is outside of higher-level care setting	Having been withheld because the patient was not receiving advanced respiratory support, advanced respiratory support is reinstituted
The patient is not receiving advanced respiratory support	Having been withheld because the APTT was unacceptably prolonged, the APTT becomes acceptable
The treating physician deems that there is a clinically unacceptable increase in APTT (>100s)	Having been withheld because there was excessive bloodstaining of respiratory secretions, the bloodstaining of the respiratory secretions has resolved
The treating physician deems that there is excessive bloodstaining of respiratory secretions	Having been withheld for suspected HIT, the patient is found not to have this condition
There is pulmonary bleeding, major bleeding or suspected or confirmed HIT	Having been withheld for ECMO, the treatment with ECMO is stopped
The patient is receiving ECMO or HFOV	Having been withheld for HFOV, the treatment with HFOV is stopped

Each participant has the right to withdraw from the trial at any time. Where the participant gives a reason for withdrawal (they are not obliged to provide a reason), the reason for withdrawal will be recorded. Equally, the investigator may decide to withdraw a patient from the study at any time — in this case, the reason for withdrawal will be recorded. If a patient withdraws from the study, they will be encouraged to complete the follow-up aspect of the study. Also, if a patient withdraws from the study due to a safety event, mandatory follow-up of that event until its resolution will occur.

#### Strategies to improve adherence to interventions {11c}

As a phase 1b/2a study, any issues that arise with adherence to intervention will be reported and will be addressed during the study. These may include staffing issues due to the pandemic or any supply or administration issues related to drug and equipment which may arise. In general, we anticipate high levels of adherence as the intervention is provided in the ICU, by ICU staff.

#### Relevant concomitant care permitted or prohibited during the trial {11d}

Patients receiving or anticipated likely to receive nebulised tobramycin are prohibited from enrolling in the trial or must be withdrawn if this therapy is commenced. Patients receiving direct oral anticoagulants are also prohibited from enrolment.

Treatment with any or all of the following therapies *is not* of itself reason to withhold nebulised heparin: deep vein thrombosis prophylaxis with unfractionated heparin or non-heparin anticoagulants, anti-thrombotic medications, protamine, prone positioning and inhaled nitric oxide.

#### Provisions for post-trial care {30}

There will be no formal provision of the intervention beyond the trial period, as recommended by the World Health Organization (WHO) interim guidance that any specific therapy targeted to SARS-CoV-2 infection should be provided only as part of a research protocol [12]. All subsequent requirement of both SARS-CoV-2 and unrelated needs will be under the existing healthcare system in the recruiting centres.

#### Outcomes {12}

The co-primary outcomes are:

1. A between-group difference in d-dimer levels over time, assessed by measurement of area under the curve (AUC) on days 1, 3, 5 and 10
2. A between-group difference in the occurrence of serious adverse events (SAEs) as listed in the safety analysis

Secondary efficacy outcomes include analysis of between-group differences in:

1. Oxygenation index (OI) = (FiO_2_ × M_PAW_)/PaO_2_ will be recorded every 6 h
2. Pulmonary compliance will be recorded every 6 h up to day 10 in invasively ventilated patients
  - Dynamic pulmonary compliance is measured automatically on our ventilators (Puritan Bennett and Servo I) and recorded on the ICU Clinical Information System), measured as ∆V/∆P
3. Inflammatory and coagulation markers
  - IL-1β, IL-6, IL-8, IL-10, soluble tumour necrosis factor receptor 1, C-reactive protein, procalcitonin, ferritin, fibrinogen and lactate dehydrogenase LDH will be assessed
  - AUC measurement for these inflammatory and coagulation markers on days 1, 3, 5 and 10
  - Ratio of IL-1β/IL-10 and IL-6/IL-10
4. Time to separation from invasive ventilation to day 28, where non-survivors to day 28 are treated as though not separated from invasive ventilation, measured in days
5. Number tracheotomised to day 28 (*N*)
6. Time to separation from the ICU to day 28, where non-survivors to day 28 are treated as though not separated from invasive care measured in days
7. Survival to day 28, survival to day 60 and survival to hospital discharge, censored at day 60

Additional outcomes include:

1. Time to separation from invasive ventilation to day 28, among survivors
2. Number treated with neuromuscular blockers instituted after enrolment to day 10
  - Any administration of neuromuscular blocking drugs will be recorded
3. Number treated with prone positioning instituted after enrolment to day 10
4. Number treated with extra-corporeal membrane oxygenation instituted after enrolment to day 10
5. Time to separation from the ICU to day 28, among survivors, measured in days
6. Number residing at home or in a community setting at day 60
7. Number residing at home or in a community setting at day 60, among survivors

Safety outcomes include analysis of between-group differences in:

1. Air quality samples from an infection control perspective (A TSI optical particle sizer device will be used to collect data on fugitive aerosol emissions during nebulisation, and throughout randomly selected 24-h periods in intubated and non-intubated patients, and in control and treatment arm patients.) This will be a descriptive analysis of air quality information in a selected number of patients.
2. Number of patients transfused red blood cells to day 10
3. Volume of red blood cells transfused to day 10
4. Number who record major bleeding to day 10
  - ‘Major bleeding’ is defined as bleeding that results in death and/or bleeding that is symptomatic and occurs in a critical area or organ (intra-cranial, intra-spinal, intra-ocular, retroperitoneal, intra-articular or intramuscular with compartment syndrome) and/or bleeding that results in a fall in haemoglobin of 20g/l or more, or results in transfusion of two or more units of whole blood or red cells [13]
5. Number of patients who record clinically relevant non-major bleeding as defined by the International Society on Thrombosis and Haemostasis [14]
6. Number who record a diagnosis of heparin-induced thrombocytopenia (HIT)
7. Number who record other adverse events and reactions
  - Adverse events will be assessed systematically with patient and chart reviews on a daily basis, with all clinical events reviewed and assessed by the investigator, and adverse events recorded and reported appropriately. AEs will be classified by organ system

#### Participant timeline {13}

*Day 0*: screening, consent/next of kin assent, enrolment and randomisation

*Day 1*: The following baseline data will be recorded on the baseline electronic case report form (eCRF):

- Eligibility criteria

- Intubation date and time and intubation setting

- Birth date, sex, height and weight

- History of tobacco smoking, hypertension, diabetes mellitus, asthma and chronic obstructive pulmonary disease

- Hospital admission date, ICU admission date and time, and acute physiology and chronic health evaluation (APACHE) III score, ICU diagnosis

-Treatment in the 24 h prior to randomisation with unfractionated heparin, intravenously or subcutaneously; LMWH, intravenously or subcutaneously; lopinavir-ritonavir; remdesivir; hydroxychloroquine; interferon-β; interleukin antagonists; oseltamivir, laninamivir, zaninamivir or peramivir; macrolide; non-macrolide antibacterial; antifungal; and corticosteroid

- Treatment at the time of randomisation with inotrope or vasopressor infusion, renal replacement therapy, neuromuscular blocker and prone positioning

- Serum creatinine and bilirubin, blood haemoglobin, white cell count and platelet count, blood activated partial thromboplastin time (APTT) and international normalised ratio (INR), collected closest to and before randomisation

- For the chest radiograph or computerised tomography (CT) of chest performed closest to and before randomisation: the number of lung quadrants with acute opacities that are not fully explained by effusions, lobar/lung collapse or nodules; whether the opacities are present bilaterally; whether, given all the medical information about the patient, the opacities are entirely attributable to cardiac failure or fluid overload; and whether there is objective evidence to exclude the possibility of cardiac failure or fluid overload

- Arterial blood gas (pH, PaCO2, PaO2, bicarbonate and lactate) and corresponding ventilation parameters (respiratory rate, FIO2, peak airway pressure, positive end expiratory pressure (PEEP) or continuous positive airway pressure (CPAP) and tidal volume) closest to and before randomisation

*Days 0 to 10*: The following data are gathered from health records at the treating institution:

- Type of respiratory support — high-flow nasal O_2_ (>30l/min)/non-invasive ventilation/invasive mechanical ventilation

- Dose of nebulised unfractionated heparin

-Treatment with unfractionated heparin, intravenously or subcutaneously; LMWH, intravenously or subcutaneously; lopinavir-ritonavir; remdesivir; hydroxychloroquine; interferon-β; interleukin antagonists; oseltamivir, laninamivir, zaninamivir or peramivir; macrolide; non-macrolide antibacterial; antifungal; corticosteroid; inotrope or vasopressor infusion; renal replacement therapy; neuromuscular blocker; prone positioning; and extra-corporeal membrane oxygenation

- Volume of packed red cells and whole blood transfused

- Highest APTT

- Blood samples will be collected for inflammatory marker and coagulation parameter assessment as per the primary and secondary outcomes, on days 0, 3, 5 and 10

*Day 28*: The following data are gathered from health records at the treating institution and, where necessary, other healthcare providers:

- Date of first diagnosis of COVID-19 (clinical diagnosis)

- Date of first positive respiratory sample for SARS-CoV-2 and the sample type

- Acute hospital discharge status at day 28 and, if discharged, the date of discharge and discharge destination including deceased

- Readmission to ICU during the acute hospital admission and prior to day 28

- ICU status at day 28 and, if not in the ICU at the end of day 28, the final date and time of ICU discharge

- Invasive ventilation status at day 28 and, if not receiving invasive ventilation at the end of day 28, the final date and time that invasive ventilation was stopped

- Tracheotomy and, if performed, the procedure date

*Day 60*: The following data are gathered from health records at the recruiting institution and, where necessary, other healthcare providers and the patient or proxy:

- Vital status at day 60 and, if deceased, the date of death and whether death related (directly or indirectly) to COVID-19 infection

- Place of residence at day 60

- If not discharged from the acute hospital discharge status by day 28, the acute hospital discharge status at day 60 is ascertained and, if now discharged, the date of discharge and the discharge destination including whether deceased.

#### Sample size {14}

This is a phase 1b/2a study aiming to evaluate the effect of nebulised unfractionated heparin on procoagulant markers and the safety of nebulised heparin in patients receiving advanced respiratory support for COVID-19 lung disease. This has not been studied previously, and as such, data does not exist to power the study accurately to assess clinical outcomes. We have chosen to base our power analysis on some of our own in house data for d-dimer levels in COVID patients who required ICU care (12 patients) and those who did not (26 patients) (unpublished data from very early in the pandemic).

Based on an estimation that nebulised heparin may reduce the d-dimer levels in ICU patients (mean = 944.8ng/ml [SD = 485.3]) to those experienced in ward patients (mean = 436.5ng/ml [SD = 604.0]), with an alpha level of 0.05 and a power of 90% to detect a type II error, 19 patients per group would be required. Increasing the number by 1 per group to allow for potential dropout gives a sample size of 20 patients per arm of the study, with a total of 40 patients to be enrolled. The safety profile of administering nebulised heparin to invasively ventilated patients with COVID-19 will be a co-primary outcome. The study is not powered for analysis of the co-primary endpoint of serious adverse events due to insufficient pre-existing data in this population. Power analysis is based only on the outcome of d-dimer as described above.

### Recruitment {15}

Recruitment will be organised and supervised by intensive care research and clinical research facilities within recruiting centres and in line with national and international standards and guidelines, and local standard operating procedures. The research nurses/coordinators and investigators at each site will work with clinicians to identify potential candidates for enrolment. A screening log will be maintained of patients who met the inclusion criteria but were not enrolled, with the reason for exclusion recorded on the log. If a patient is deemed eligible, the patient’s next of kin will be contacted, and a patient information leaflet will be sent to them or given to them in person if possible.

### Assignment of interventions: allocation

#### Sequence generation {16a}

Allocation will be carried out after consent is confirmed, via a central, secure web randomisation process hosted by the contract research organisation (CRO). Allocation will be in a one-to-one ratio, with variable block size randomisation. The code for the randomisation process is embedded in the eCRF. The Programme R was used to generate the randomisation sequence.

#### Concealment mechanism {16b}

Blocks of variable size and a random seed will be used to ensure allocation concealment cannot be violated by deciphering the sequence near the end of each block. To further protect from deciphering, block size will not be revealed to site investigators. Site-level randomisation will be used. At randomisation, each participant is assigned to nebulised heparin or standard care. There is a one-to-one allocation ratio.

#### Implementation {16c}

Research nurses and investigators at each site will work with clinicians to identify potential candidates for enrolment. The randomisation process is embedded in the eCRF, and once the patient’s screening data has been inputted and they are eligible, the investigator will request randomisation via the eCRF.

### Assignment of interventions: blinding

#### Who will be blinded {17a}

This is an open-label trial, and therefore, all patients facing study personnel will be unblinded. However, the statistician performing data analysis will be blinded to allocation.

#### Procedure for unblinding if needed {17b}

Not applicable—this is an open-label trial.

### Data collection and management

#### Plans for assessment and collection of outcomes {18a}

Data will be collected by trained staff at each site under the supervision of the principal investigator using a case report form and data dictionary developed by the management committee (Attachment CRF). Baseline data will be gathered from health records at the treating institution. It may also be necessary in a small number of cases to obtain information from other healthcare providers. Daily data for days 0 to 10 while in intensive care: Data are gathered from health records at the treating institution for each day that the patient is in ICU up to and including day 10. Day 28 and day 60 data are gathered from health records at the treating institution and, where necessary, other healthcare providers. Adverse event or adverse reaction data are gathered from health records at the recruiting institution and, where necessary, other healthcare providers and the patient or proxy.

The study will be conducted in accordance with the current approved protocol, International Conference on Harmonisation (ICH) Good Clinical Practice (GCP), relevant regulations and standard operating procedures. All effort shall be taken to ensure that data collected is accurate, complete and reliable. The site must facilitate all required monitoring and respond to queries in a timely manner. All data will be reviewed for completeness and logical consistency by the data management team. Data queries will be generated via the electronic data capture system to correct or clarify data or request missing information. The designated site staff will be required to respond to these queries in accordance with data entry and data query timelines for the study.

#### Plans to promote participant retention and complete follow-up {18b}

N/A — participation by patient is only required while in ICU. Follow-up requires using healthcare records and information from primary care providers only. This is an intention-to-treat study design.

#### Data management {19}

All electronic information held will be kept on a password-protected, audited server maintained by the CRO, accessible only to authorised study personnel and compliant with ICH GCP.

All study material, including case report forms and the study database, will be stored for a minimum period of 15 years after the conclusion of the study or as per national/European legislation.

Source documents are the documents or media where the patients’ data are first recorded, and from which the data participants’ entered into the eCRF data is obtained. These include, but are not limited to, data gathered from health records at the treating institution, medical records from other institutions where applicable.

Sources of data will be identified and pre-specified on a study-specific source data agreement relevant to the study site. The source data agreement will be verified by the principal investigator with a copy of the agreement to be filed within the site file.

The site investigator(s) will facilitate the permit of study-related monitoring, audit, research ethics committee approval review and regulatory inspection by the relevant national authority/international, providing direct access to source documents where possible.

Data collected will be pseudo-anonymised; on all study-specific documents other than the signed consent, the subject will be referred to by the study subject identification number/code.

Subject participation and subject progress should also be recorded in the subject medical records to ensure relevant healthcare providers have knowledge of the subject’s participation in the study.

Data from medical records will be entered by authorised and delegated site personnel into the electronic case report form. The data will be verifiable against original records and source notes by the Monitor during monitoring visits. Data reported on the eCRF that are derived from source documents must be consistent with the source documents or the discrepancies must be explained.

eCRF completion instructions will be provided and any questions about recording specific information on the eCRF should be directed to the investigator. Each participating centre will maintain a subject identification log of enrolled patients that includes patient identifiers. Patient identifiers are not transferred to the study coordinating centre.

#### Confidentiality {27}

Only authorised study staff will be permitted access to the password-protected study database where study-specific data will be stored. Paper documents will be stored in a locked site with restricted access. On all trial-specific documents, other than the signed consent form and the enrolment log, the participant will be referred to by the unique participant code, not by name. Patient identifiable data will not leave the site, as per the Data Protection Act. Access will be granted to authorised representatives from the sponsor and the regulatory authorities to permit trial-related monitoring, audits and reports.

Subject confidentiality will be maintained at all times and no documents containing the subject’s name or other identifying information will be collected by the sponsor on the study database. It may be necessary for the sponsor’s representatives, the ethics committee and regulatory authority representatives to have direct access to the subject’s medical records.

#### Plans for collection, laboratory evaluation and storage of biological specimens for genetic or molecular analysis in this trial/future use {33}

Blood samples collected for analysis of interleukin and tissue necrosis factor levels will be stored during the study in the recruiting hospital in temperature monitored laboratory refrigerators. Following completion of recruitment and sample collection, samples will be transferred by a specialist biochemical sample transport company to the primary centre (NUIG/UHG) for processing. This approach has been taken as some of the inflammatory cytokine analyses are not standard assays in the laboratory of the recruiting centres. Only the described analyses will be carried out on these samples, with unused portions of these samples destroyed according to standard laboratory SOPs.

### Statistical methods

#### Statistical methods for primary and secondary outcomes {20a}

Demographic and baseline characteristics of the study population will be summarised using appropriate graphical and numerical summaries for each treatment group and by site. The primary analysis approach will be an intention-to-treat analysis, with a per-protocol analysis also being carried out. The per-protocol analysis will include any patient who received any dose of the study drug in the treatment group and any patient who underwent data collection for at least one time point in the standard care group. A safety analysis set will be included including all recruited patients. For effectiveness analysis, we will use the 2-sample *t*-tests (or suitable non-parametric test depending on data distribution) for the between-group difference in d-dimer levels and generalised linear (or non-linear as appropriate) mixed effects models for the analysis of longitudinal data, using appropriate link functions depending on the type of outcome variable. This analysis will be used to compare the treatment effect, while adjusting for patient characteristics (e.g. Horowitz index of lung function (P/F ratio), Sequential Organ Failure Assessment (SOFA) score, body mass index (BMI), sex and age) as appropriate for all primary and secondary efficacy outcomes. A Cox proportional hazards model will be used to model time to event responses. Generalised linear models will be used for the analysis of safety outcomes. Primary and secondary outcome data will be suitable for meta-analysis to further assess clinically relevant outcomes. Interval estimates (95% confidence intervals) for the treatment effect will be reported accordingly.

#### Interim analyses {21b}

There is no plan to carry out an interim analysis as the primary outcome is not a clinical outcome, and the numbers recruited are small.

#### Methods for additional analyses (e.g. subgroup analyses) {20b}

Results will be presented as per-protocol and intention-to-treat analysis groups. Results will also be presented for ventilated at enrollment and non-ventilated at enrollment subgroups, although due to the sample size the numbers in each group will be small.

#### Methods in analysis to handle protocol non-adherence and any statistical methods to handle missing data {20c}

All participants who are enrolled will be included in the analysis. Any participants who withdraw and wish to have their data withdrawn will not be included. Analysis will be carried out using the intention-to-treat principle where patient’s data will be analysed as members of the group they were randomised to, irrespective of their compliance with the treatment as specified in the protocol. A separate per-protocol analysis will be carried out including all patients who complied with the protocol in terms of exposure to planed treatment, availability of measurements and absence of major protocol deviations. Any deviation(s) from the original statistical plan will be described and justified in the final report. [An analysis of all missing data will be carried out to identify the likely missing data mechanism (e.g. missing completely at random, missing at random, and missing not at random) and to investigate the reasons for missingness as a useful outcome for the corresponding subsequent definitive trial.] Multiple imputation using chain equations will be used to handle missing data (due to outcome values missing in some visits for some of the participants) in the primary and secondary outcome analysis.

#### Plans to give access to the full protocol, participant-level data and statistical code {31c}

The full protocol is available on application to the investigators, with the summary protocol published here. The statistical code will be published as an addendum to the results paper after completion of the study. Participant-level data will be maintained confidentially, with some clinical outcome data feeding into the CHARTER and INHALE-HEP international meta-trials [15, 16]. Reasonable requests for access to the data from other investigators will be considered by the investigators, taking into account General Data Protection Regulation (GDPR) compliance.

### Oversight and monitoring

#### Composition of the coordinating centre and trial steering committee {5d}

The Health Research Board Clinical Research Facility (HRB-CRFG) Galway will be the coordinating centre, taking overall responsibility for the conduct of the trial. All members of the investigator team and research group will have appropriate GDPR and ICH GCP training, which will be documented in a delegation log. Regular meetings between each site and the coordinating centre, facilitated by the CRO, will allow communication of progress, highlighting of adverse reactions and discussion of technical issues. A detailed plan of sponsor oversight with regular site audit has been devised.

#### Composition of the data monitoring committee, its role and reporting structure {21a}

An independent international Data Safety Monitoring Committee (DSMC) will oversee the conduct of the trial and verify the correct implementation of the clinical trial design. Just as with a traditional trial design, the role of the DSMC is to ensure the design is implemented with integrity, that the original design remains scientifically and ethically appropriate in light of the accumulating data and any external information, and to protect research participants from avoidable risk. The DSMC for this trial will include expertise in critical care, biostatistics and clinical trials.

The responsibilities of the DSMC are to advise on all matters related to the safety of subjects enrolled in this study, the integrity of trial data and conclusions and the appropriateness of continued trial conduct.

The aims of the DSMC include:

1. To identify any trends, such as increases in un/expected events, and take appropriate action
2. To seek additional advice or information from investigators where required
3. To evaluate the risk of the trial continuing and take appropriate action where necessary

The researchers will provide the DSMC with an interim report of adverse events and early results. The DSMC will also undertake a review of enrolments and withdrawals according to the DSMC Charter, to ensure adequate study safety, and minimal risk to participants. Where necessary, the DSMC will make recommendations for corrective action which may include early termination, suspension or modification of a trial, or changes in consent processes. However, the final decision will rest with the sponsor.

### Adverse event reporting and harms {22}

#### Procedures for recording adverse events

Reportable adverse events and reactions will be communicated by site investigators to the chief investigator. In general, this will occur in writing within no more than 3 days of the site investigator becoming aware of the event. The management committee will assess all safety reports received from investigators.

All adverse events occurring during the trial that are observed by the investigator or reported by the participant will be recorded on the eCRF, whether or not attributed to trial medication.

The following information will be recorded: description, date of onset and end date, severity, assessment of relatedness to trial medication, other suspect drugs or devices and action taken. Follow-up information should be provided as necessary. Only non-serious adverse events which are considered related to the investigational medicinal product will be recorded routinely on the adverse event eCRF from the time of informed consent (or enrolment into the study) up to 96 h after the last dose of study drug has been received. All adverse events meeting ‘serious’ criteria should be recorded on the SAEs CRF as per Section below.

#### Procedures for recording and reporting SAEs

All SAEs must be reported on the SAE reporting form to the sponsor within 24 h of the study team becoming aware of the event.

The sponsor will perform an initial check of the report, request any additional information and ensure it is reviewed by the Medical Monitor on a weekly basis.

Adverse events meeting the definition of SAEs must be reported using the SAE report form located in the investigator site file. All adverse events meeting ‘serious’ criteria occurring in each patient should be reported from the time of informed consent (or enrolment into the study) up to 90 days after the last dose of study drug has been received. All SAE information must also be recorded on the SAE form within the eCRF. Additional and further requested information (follow-up or corrections to the original case) may be captured within the SAE form and eCRF. Follow-up/new information is required within the same reporting timeline, i.e. within 24 h of the study team becoming aware of the new information.

SAEs will also be reviewed by the DSMC, in accordance with their Charter.

Sponsor responsibilities:

The sponsor, National University of Ireland Galway (NUIG), will delegate appropriate adverse event reporting for the study according to the applicable regulatory guidelines.

NUIG will submit local suspected unexpected serious adverse reactions (SUSARs) on an expedited basis to the local regulatory authority and other Competent Authorities involved, concerned Ethics Committees, all participating investigators and the DSMC. NUIG sends annual Development Safety Update Reports to the concerned regulatory authority and Ethics Committee. Investigators will be informed of SUSARs.

For fatal and life-threatening SUSARS, this will be done no later than 7 calendar days after the sponsor is first aware of the reaction. Any additional relevant information will be reported within 8 calendar days of the initial report. All other SUSARs will be reported within 15 calendar days.

### Frequency and plans for auditing trial conduct {23}

The trial will be conducted in accordance with the current approved protocol, GCP, relevant regulations and standard operating procedures. Regular monitoring will be performed according to GCP and as defined within the study Monitoring Plan. The CRO will conduct site initiation visits, on-site monitoring visits, remote monitoring, eCRF data reviews and close out visits at all sites. The University Hospital Galway Clinical Research and Development Office will also conduct an independent full audit of the study. These monitoring visits are independent of the investigators and the sponsor. Data will be evaluated for compliance with the protocol and accuracy in relation to source documents. The Monitor will perform source document verification to verify that the clinical trial is conducted and data are generated, documented and reported in compliance with the protocol, GCP and the applicable regulatory requirements.

A quality assurance audit/inspection may be conducted by the competent authority, sponsor or an agent delegated responsibility.

Data will also be monitored by the DSMC in accordance with the DSMC Charter.

### Plans for communicating important protocol amendments to relevant parties (e.g. trial participants, ethical committees) {25}

Protocol amendments will be reviewed for their impact as per requirements and all substantial amendments will not be implemented prior to pre-approval by the competent authority and the ethics committees. Protocol amendments will be updated on relevant clinical trial registries by an appropriate delegated person the sponsor or the sponsors representative.

If a protocol amendment requires a change to the informed consent form or any relevant document, the sponsor or their delegate will ensure that all related documents will be updated or considered for update, and all approvals sought as required.

### Dissemination plans {31a}

The study site(s), represented by the primary investigator, will take responsibility to report the results in a scientific peer reviewed journal, according to the International Committee of Medical Journal Editors recommendations. Results of the study will be sent to participants on request (once available) and will be made available on a publicly available trial registry website, recognised by the World Health Organization International Clinical Trials Registry Platform (WHO ICTRP) as a primary registry. Authorship of any publication of results will include investigators and collaborators involved in the study according to the ICJME criteria.

## Discussion

Nil extra.

## Trial status

The CHARTER-Ireland trial commenced December 23, 2020, at University Hospital Galway, Ireland. Recruitment is proceeding using protocol version 7, 22 February 2021. We expect to complete recruitment in Spring 2022.

## Supplementary Information

**Additional file 1: Table S1.** Participant timeline {13}

## Data Availability

The data generated by this study will be considered confidential by the investigator, except to the extent that it is included in a publication as agreed in the publication policy of this protocol. Individual requests for access to dataset will be considered in discussion with the local research ethics committee.

## Abbreviations

NUIG: National University of Ireland, Galway
ARDS: Acute respiratory distress syndrome
sTNFR1: Tumour necrosis factor receptor 1
IL: Interleukin
ICU: Intensive care unit
APTT: Activated partial thromboplastin time
LDH: Lactate dehydrogenase
HIT: Heparin-induced thrombocytopenia
ECMO: Extra-corporeal membrane oxygenation
HFOV: High-frequency oscillatory ventilation
DVT: Deep vein thrombosis
LMWH: Low molecular weight heparin
WHO: World Health Organization
OI: Oxygenation index
Fi02: Fraction of inspired oxygen
M_PAW_: Mean airway pressure
PaO_2_: Partial pressure of arterial oxygen
AUC: Area under the curve
eCRF: Electronic case report form
INR: International normalising ratio
CT: Computerised tomography
PaCO2: Partial pressure of carbon dioxide
PEEP: Positive end expiratory pressure
CPAP: Continuous positive airway pressure
ICH: International Conference on Harmonisation
GCP: Good Clinical Practice
P/F ratio: Horowitz index of lung function
SOFA: Sequential Organ Failure Assessment
BMI: Body mass index
GDPR: General Data Protection Regulation
CRO: Contract research organisation
HRB: Health Research Board
DSMC: Data Safety Monitoring Committee
SAEs: Serious adverse events
SUSARs: Suspected unexpected serious adverse reactions
