# Supplementary material for: The CHARTER-Ireland trial: can nebulised heparin reduce acute lung injury in patients with SARS-CoV-2 requiring advanced respiratory support in Ireland: a study protocol and statistical analysis plan for a randomised control trial
Source: Trials. 2022 Sep 14;23:774. doi: 10.1186/s13063-022-06518-z (PMC9471050; doi:10.1186/s13063-022-06518-z)
Supplement: Supplementary file 1 — Additional file 1: Table S1. Participant timeline {13} [file 13063_2022_6518_MOESM1_ESM.doc]

Table 1. Participant Timeline {13}

|  | **STUDY PERIOD** | | | | | | | |
| --- | --- | --- | --- | --- | --- | --- | --- | --- |
|  | **Enrolment** | **Allocation** | **Post-allocation** | | | | | **Close-out** |
| **TIMEPOINT**** | ***-1D/0D*** | **0D** | ***1D*** | ***3D*** | ***5D*** | ***10D*** | ***28D*** | ***60 D*** |
| **ENROLMENT:** |  |  |  |  |  |  |  |  |
| **Eligibility screen** | X |  |  |  |  |  |  |  |
| **Informed consent** | X |  |  |  |  |  |  |  |
| **Allocation** |  | X |  |  |  |  |  |  |
| **INTERVENTIONS:** |  |  |  |  |  |  |  |  |
| ***Nebulized Unfractionated heparin*** |  |  |  |  |  |  |  |  |
| ***Standard of Care*** |  |  |  |  |  |  |  |  |
| **ASSESSMENTS:** |  |  |  |  |  |  |  |  |
| ***Demographic, co-morbidities*** | X |  |  |  |  |  |  |  |
| ***History of current admission*** | X |  |  |  |  |  |  |  |
| ***Hemoglobin, platelets, liver and kidney function*** | X |  |  |  |  |  |  |  |
| ***APACHE III*** | X |  |  |  |  |  |  |  |
| ***Treatment with Anti-coagulants/***  ***Anti-biotics/ immunomodulators*** | X |  | X | X | X | X |  |  |
| ***Ventilatory +organ support*** | X |  | X | X | X | X |  |  |
| ***Arterial blood gas + Ventilatory parameters (6 hourly)**** | X |  | X | X | X | X |  |  |
| ***D-Dimer*** |  |  | X | X | X | X |  |  |
| ***Coagulation markers*** |  |  | X | X | X | X |  |  |
| ***Inflammatory markers*** |  |  | X | X | X | X |  |  |
| ***Red blood cells transfused*** |  |  | X | X | X | X |  |  |
| ***Highest APTT*** |  |  | X | X | X | X |  |  |
| ***Reporting of severe adverse events*** |  |  |  |  |  |  |  |  |
| ***Total ICU days*** |  |  |  |  |  |  | X |  |
| ***Total invasive ventilation days*** |  |  |  |  |  |  | X |  |
| ***Total tracheostomized days*** |  |  |  |  |  |  | X |  |
| ***Vital status*** |  |  |  |  |  |  | X | X |
| ***Date of discharge*** |  |  |  |  |  |  | X | X |
| ***Current residence*** |  |  |  |  |  |  | X | X |

*6 hourly parameters are collected each day of treatment / while the patient meets recruitment criteria.
